# Photocatalytic Degradation of Dielectric Mineral Oil with PCBs Content Coupled with Algae Treatment

**DOI:** 10.3390/toxics10050209

**Published:** 2022-04-22

**Authors:** Andrés F. Suárez, Carlos E. Camargo, Miguel A. Esteso, Carmen M. Romero

**Affiliations:** 1 Departamento de Ingenieria, Universidad de Bogota Jorge Tadeo Lozano, Bogotá 111711, Colombia; andresf.suareze@utadeo.edu.co; 2 Departamento de Quimica, Universidad Nacional de Colombia, Bogotá 111321, Colombia; carlosernestocamargo@gmail.com; 3 Universidad Catolica de Avila, Calle los Canteros s/n, 05005 Ávila, Spain; 4 Universidad de Alcala, U.D. Quimica Fisica, 28805 Alcala de Henares, Spain

**Keywords:** polychlorinated biphenyls, pollutants, photocatalytic oxidation, biotreatment, *Nostoc* sp. microorganism

## Abstract

Insulating oil contaminated with polychlorinated biphenyls (PCBs) is an environmentally important pollutant. This research focused on the establishment of the optimum conditions under which photocatalytic oxidation can be used together with biotreatment using the *Nostoc* sp. microorganism to degrade PCBs present in used dielectric oils. Among the optimal conditions studied were PCB concentration, initial pH, and titanium dioxide (TiO_2_) concentration for the photocatalytic step, and PCB concentration and photoperiod for the biotreatment step. The results indicate that the optimal conditions necessary for photocatalytic degradation were a pH of 6.10, 113 mg/L TiO_2_, and 765 mg/L PCBs, achieving close to 90% removal. For the biotreatment step, the results showed that PCBs progressively inhibited the microbiological growth, with the lowest cellular growth observed in the medium with the highest PCB concentration.

## 1. Introduction

Used oils are considered hazardous waste according to the Basel Convention. For this reason, policies and actions have been developed aimed at preventing the generation and final disposal of used oils, to conserve the natural resources and to guarantee the health of the population. However, it is presumed that, due to ignorance of technical procedures, lack of regulations and consumption standards, and the black market existing with these products, the handling given to a large quantity of used oils is inadequate, which inevitably translates into adverse effects on human health and unfavorable impacts on the environment [1,2,3,4].

Pollution generated by oils affects most natural resources, with its effect on water, air, and soils being especially harmful. As a result of being used, dielectric oils can become contaminated by physical or chemical impurities generated from their handling, storage, and processing. Additionally, used dielectric oils may contain synthetic substances of high risk to humans and the environment, such as polychlorinated biphenyls (PCBs), which have toxic properties, are persistent to degradation, bioaccumulate, and are transported by air, water, and migratory species across international borders and deposited far from the place of their release, accumulating in terrestrial and aquatic ecosystems according to the Stockholm Convention as Persistent Organic Pollutants (POPs) [5,6]. These substances have been found in various parts of the world, including in isolated areas far from those where they were used, in addition to human settlements, and even in breast milk [7]. Due to their nature, POPs (which are well-known carcinogens) are difficult to degrade, and biodegradation, which for many pollutants is the main elimination mechanism, is limited due to their stable chemical structure, their xenobiotic nature, their low solubility in water, and their strong sorption in the soil [8,9].

On the other hand, the contamination generated by this class of compounds affects the majority of natural resources; in water, for example, used oils form a film that produces a separation between the air and the aqueous phases, preventing the oxygen contained in the air from dissolving in water [10,11,12]; in the soil, the used oils produce an alteration of the humus and the possible post-contamination of surface and underground waters; in the air, the combustion of contaminated dielectric oils when used as fuels or as fuel–oil mixtures generates toxic compounds to be emitted into the atmosphere; even the ashes produced by these processes may be contaminated with heavy metals such as lead, chrome, and cadmium [13,14,15].

**Figure 1:**
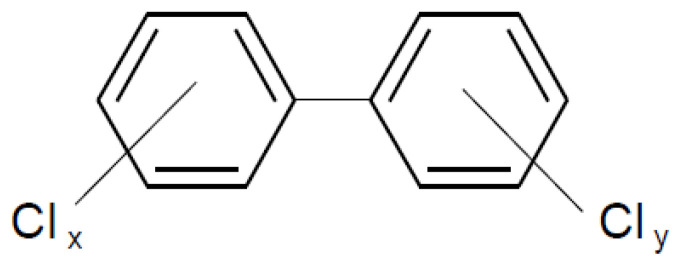
General molecular structure of PCBs.

Polychlorinated biphenyls (PCBs) are aromatic, synthetic chemicals with the formula C_12_H_10−_nCl_n_, where n ranges from 1 to 10. The term polyhlorinated biphenyls has been used to refer to a set of halogenated aromatic compounds of high thermal stability composed of two benzene rings substituted with chlorine atoms, joined by a bond between carbon and carbon. Figure 1 shows the general molecular structure of PCBs.

PCBs can be obtained via different synthetic routes, generally by direct chlorination of aromatic substrates forming nonsymmetric chlorobiphenyls, by aryl condensation reactions to produce symmetric chlorobiphenyls, and by direct substitution on a developed biphenyl system. These compounds are not very corrosive and have very low flammability. For this reason, they have been used since the beginning of the 20th century as dielectric and heat exchange fluids, as well as additives in oils, sealants, inks, paper, paints, and coolants, among a wide variety of applications. PCBs generally have a colorless or light-yellow oily liquid appearance, with no odor or taste [7,8]. Due to their high boiling point, low dielectric constant, high solubility in organic solvents, and high stability, they have been used as dielectric fluids in electrical transformers and condensers [16,17,18,19,20,21,22,23,24,25,26].

There are several methods that have been used to treat and remove organic pollutants such as PCBs. Degradation methods include biodegradation, physical, biological, and chemical treatments such as photocatalysis, and oxidation processes.

Heterogeneous photocatalysis is a clean and low-cost method in which a light-induced transformation occurs in the presence of a catalyst. It has been considered an important technique to degrade industrial effluents and to remove heavy metals and hazardous substances, since the resulting products are generally nontoxic [24,25,27,28,29].

The heterogeneous photocatalysis of molecules such as PCBs depends on parameters such as the intensity of UV radiation, nature, concentration of the catalyst, and initial pH. In the literature, there are several reports concerning experimental conditions used for the degradation of PCBs by heterogeneous photocatalysis; however, the optimal conditions depend on the type of the waste products that contain the organic pollutants [30,31,32,33,34]. TiO_2_ has been widely used as a photocatalyst because it is easily available, inexpensive, and nontoxic, with high chemical stability. Previous studies on the loss of catalyst efficiency showed that the catalyst slowly loses its ability to adsorb and degrade the contaminant mixture [28,34,35].

Biotreatments are processes that have been used to treat contaminated media, resulting in the decomposition of organic substances, including organic pollutants, by living organisms. In these methods, microorganisms can be aerobic or anaerobic; as a result, nontoxic compounds with different characteristics are produced. They are less expensive and sustainable; however, the processes are slow and, for this reason, they have been used in combination with other techniques [36,37,38].

In this work, the removal of PCBs from used dielectric oils was studied, through the use of photocatalytic treatment with TiO_2_ as the photocatalytic material, coupled to biotreatment through the cultivation of *Nostoc* sp. to propose an alternative for the degradation of these persistent organic compounds. 

The need to reduce the environmental impacts generated by natural resources has driven the continuous innovation of techniques aimed at generating better use of hazardous and persistent pollutants organic waste. Therefore, this research aims to incorporate advances in the treatment of oils in such a way that treatment alternatives are presented in the environmental and biological field. The feasibility of implementing used oil treatment processes and the benefits that these processes can represent for human health and the environment are important. Better use of these oils will translate into important environmental, economic, and social benefits, by providing new alternatives to achieve the removal of contaminants.

## 2. Materials and Methods

The dielectric oil sample used in this project came from disused equipment in the electricity sector and was obtained thanks to the support of the private company LITO SA, a Colombian organization dedicated to the management of industrial surpluses and hazardous waste. This sample was collected and stored in suitable containers to prevent contamination and deterioration in the laboratories of Universidad Nacional de Colombia.

The strains of the *Nostoc* sp. species were obtained thanks to the support of the Microalgae Culture Laboratory of Jorge Tadeo Lozano University.

Identification of PCBs was carried out following the analytical conditions to determine PCB congeners described by Wong [39], using a Shimadzu gas chromatograph–mass spectrometer GC–MS-QP2010 with helium as the carrier gas at a flow of 1.4 mL/min, and an HP-5 MS column 5% in phenyl methyl silicone following a temperature program starting at 50 °C for two min, changing to 160 °C at 10 °C/min, increasing to 190 °C at 1 °C/min, and increasing to 270 °C at 2 °C/min, employing a quadrupole mass spectrometry detector at 280 °C.

The PCB content was carried out following method 8280a of the US EPA [40], establishing the total content of PCBs in extracts prepared in cyclohexane based on Aroclor 1016 using a Hewlett Packard 6890 gas chromatograph equipped with an ECD electron capture detector and a DB-5 column; the injection was performed in splitless mode.

Heterogeneous photocatalysis was carried out in a glass reactor equipped with a cooling jacket and an OSRAM power star HQI TS 150W/WDL UV fluorescent tube using as a catalyst titanium dioxide TiO_2_ (P-25 non-porous powder, porous 80% anatase, average diameter 0.02 mm, specific surface area 50 m^2^/g) and as a surface active agent the commercial product nonylphenol ethoxylate 10 M in a concentration of 40 mg/L; the exposure time to UV radiation was 120 min, and photocatalysis was carried out with constant agitation at 6000 rpm.

To determine the optimal conditions in which heterogeneous photocatalysis could be carried out, a Box–Behnken experimental design was proposed, having as variables the initial concentration of PCBs, catalyst concentration, and initial pH and as response variables the removal of total organic carbon, the removal of the total content of PCBs, the concentration of chloride ions, and the electrical conductivity. To make the adjustments to pH 3.0 and 11.0, 10% sulfuric acid and 10% potassium hydroxide were used, while, to adjust to pH 7.0, 1% sulfuric acid and 1% potassium hydroxide were used.

The determination of the chloride content was carried out using potentiometric titration with silver nitrate at a concentration determined by a standard solution of sodium chloride, using an Automatic Titrator Metrohm 904 Titrando 815 Robotic Processor employing a Metrohm combined electrode 3.0430 100 Ag-titrode.

The conductivity of the samples was determined in triplicate in a Sartorius Professional Meter PP-50 cell B112806006 Conductivity/ATC 4-bank conductivity meter, using as calibration material KCl standard solutions of concentrations of 0.1, 0.01, and 0.001 M. 

The pH of the samples was determined in triplicate using a combined glass membrane pH electrode in a Metrohm pH 6.0259.100 bulb coupled to a Metrohm 780 pH meter and a Metrohm 6.11110.100 Pt 1000/B/2 temperature probe, using as reference material Metrohm buffer solutions of pH 4.0, 7.0, and 10.0.

Microalgae tests were carried out in sterilized glass containers. The exposure time was 7 days. They were carried out in duplicate at an interval of 15 days. In each container, an initial *Nostoc* sp. concentration of 2.0 × 10^5^ cells/mL of culture medium was inoculated.

The conditions of the experiments varied according to a factorial experimental design having as variables the photoperiod and concentration of PCBs from the photocatalytic effluent at optimal conditions. For this purpose, the concentration of 0 mg/L PCBs was considered as the control treatment, subsequently determining as growth variables the cell density expressed as the count in the Neubauer chamber by extrapolation of the absorbance at 665 nm, the change in the content of total organic carbon of the extract, and the content of chlorophyll *a* according to the formula of Strickland and Parsons for the corresponding extracts in acetone at 90% [41].

**Figure 2:**
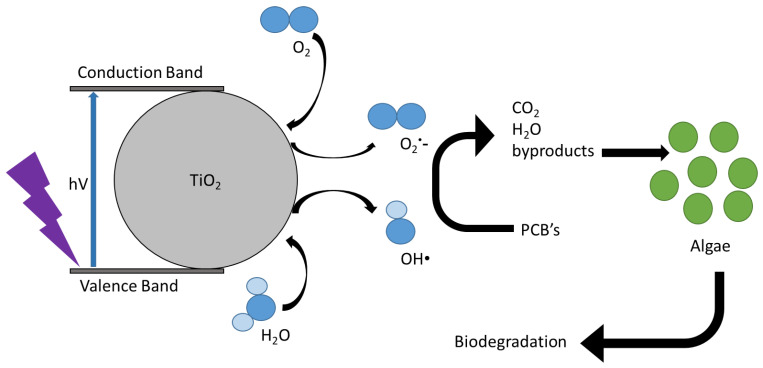
Schematic illustration for the photocatalytic treatment coupled to the biotreatment process of dielectric oils.

A schematic illustration of the photocatalytic treatment coupled to the biotreatment process of dielectric oils is presented in Figure 2.

## 3. Results and Discussion

The volatile compounds of the dielectric oil samples were qualitatively analyzed by gas chromatography–mass spectrometry using hexane R.A as solvent in a dilution of 1 to 1000. Table 1 compiles the results obtained. The gas chromatography analysis of the oil allowed the identification of 23 species of PCBs as most of the volatile compounds present in the sample.

The analysis of the mass spectra confirmed the presence of peaks corresponding to ions characteristic of the breakdown of compounds such as PCBs. For example, in the case of the molecular ion peak of peak number 12 in the chromatogram, an *m*/*z* ratio close to 257 was recorded, estimated as the mass loss corresponding to the successive loss of chlorine atoms; thus, for the molecular ion peak –1Cl, *m*/*z* was 222, and, for the molecular ion peak –2Cl, *m*/*z* was 187. This information led to the identification of this compound as 3,4,4′-trichloro-1,1′-biphenyl.

**Figure 3:**
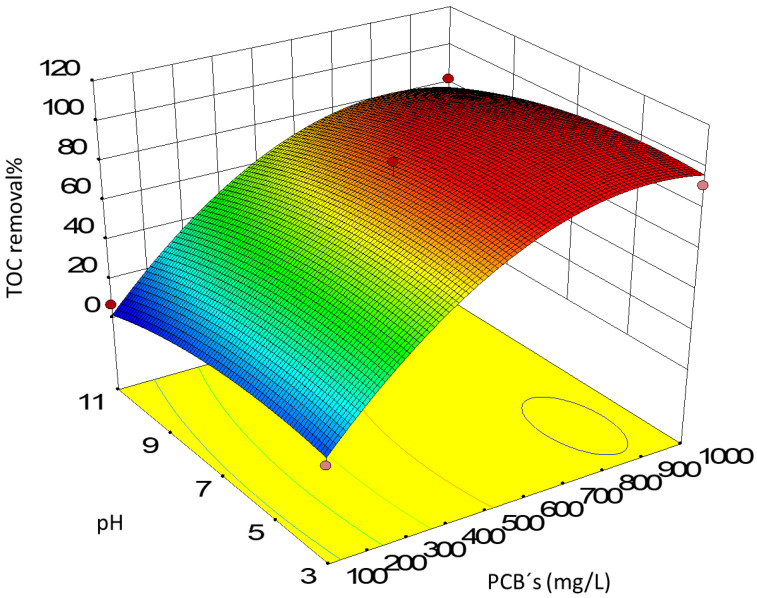
Surface response for the TOC removal of the photocatalytic experiments.

The results of total organic carbon removal of the proposed Box–Behnken experimental design are summarized in Figure 3.

As a solution for the optimum of this response surface, the values suggested by the Design Expert 10 software were taken; they corresponded to a pH of 5.6, a concentration of titanium dioxide of 262 mg/L, and a concentration of PCBs of 790 mg/L.

**Figure 4:**
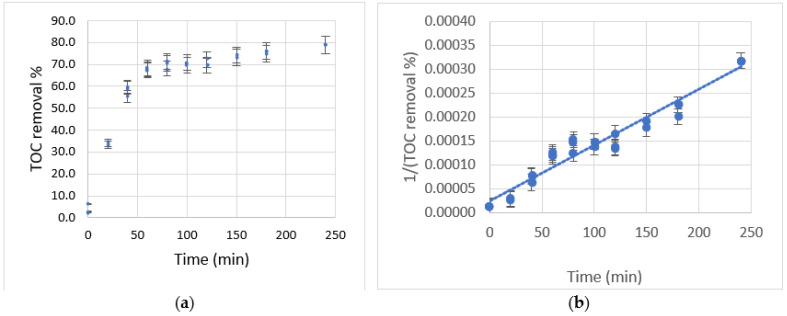
(**a**) TOC removal percentage vs. time for the optimal of the SRM; (**b**) van’t Hoff adjustment of PCB removal data.

Starting from these values as reference conditions, the corresponding photocatalytic experiment was carried out to determine the percentage of total organic carbon removal and its behavior as a function of time. The results obtained are shown in Figure 4a.

The results show an accelerated removal in the first hour of reaction until reaching values close to 70%, where the speed of removal decreased slowly until reaching removals close to 80% in a reaction time of 4 h. For the proposed time of 120 min, a removal level of around 70% was reached, indicating that the adjustment is an acceptable representation of the process. The main reason for the decrease in total organic carbon removal is the depletion of the carbon source.

By performing a van’t Hoff type kinetic fit, it was possible to establish a slope for the behavior of total organic carbon removal as a function of time of 3.2, suggesting pseudo-third-order reaction kinetics concerning this parameter. In this way, when plotting the inverse of the square of the total organic carbon as a function of time, a linear behavior would be expected as shown in Figure 4b. However, the value of *R^2^* obtained of 0.9527 for the dispersion of data above and below the fit suggests that the kinetics of this reaction is probably higher, is more complex, and may involve different stages and reaction steps.

**Figure 5:**
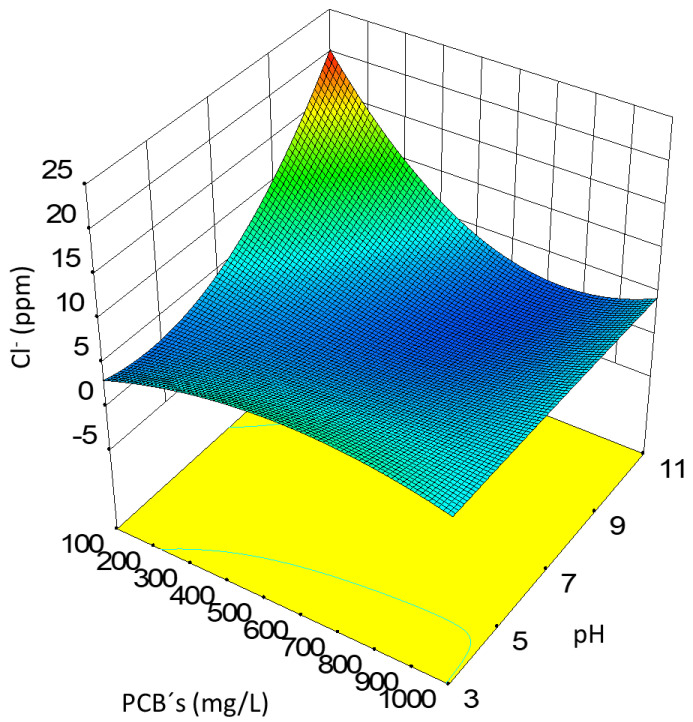
Chloride ion concentration surface response.

The results of the different potentiometric titrations of the samples proposed in the experimental design of photocatalysis expressed as chloride ion concentrations are shown in Figure 5.

The value of *R^2^* obtained of 0.9732 indicates that the combined quadratic regression adjustment was adjusted to the variation in the concentration of chlorides, while the adjusted *R^2^* statistic, which is the most adequate to compare the adjustments with a different number of independent variables, was 0.8929; using the regression coefficients obtained through the adjustment, it was possible to construct the equation of the adjusted model significantly representing the data obtained as observed through the ANOVA analysis.

When performing the analysis of the data through the combined quadratic fit for the chloride concentration, it was found that this concentration was maximum at basic pH and at a concentration of total PCBs below 500 mg/L; starting from this base and using the Design Expert 10 software, the response surface of the experimental design was determined.

**Figure 6:**
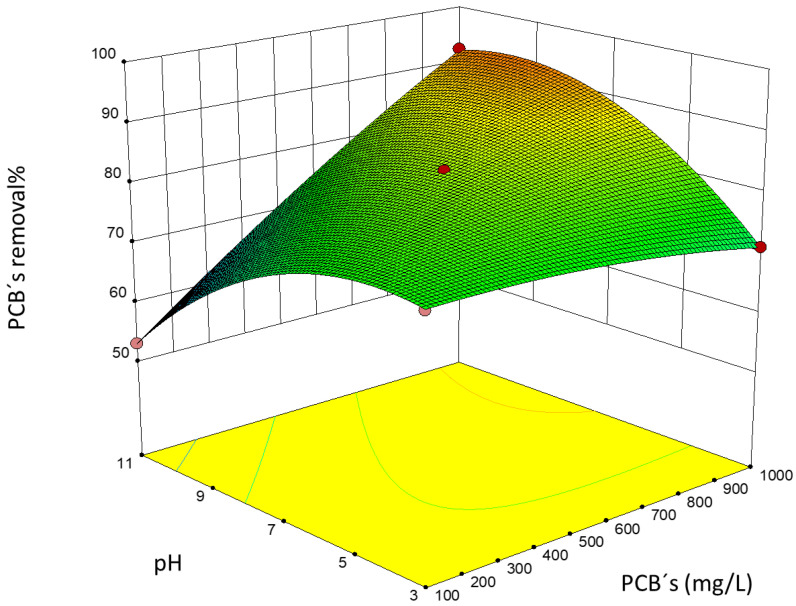
Surface response for PCB removal.

The response surface of the experimental design is shown in Figure 6.

The value of *R^2^* obtained of 0.9999 indicates that the quadratic regression adjustment fits the variation of the removal of PCBs, while the adjusted *R^2^* statistic, which is the most adequate to compare the adjustments with a different number of independent variables, was 0.9993; using the regression coefficients obtained through the adjustment, the equation of the fitted model could be constructed significantly representing the data obtained as observed through the ANOVA analysis.

When performing the analysis of the data through the quadratic adjustment for the removal of PCBs, it was found that removals greater than 90% were achieved in the maximum of the surface. Starting from this base and using the Design Expert 10 software, the optimization of the equation of the mathematical adjustment was conducted to find the conditions of pH, the concentration of PCBs, and concentration of titanium dioxide in which an optimal removal of PCBs was achieved. Five possible solutions were obtained; they ranged between pH 6.09 and 7.58, with a titanium dioxide concentration between 112 and 262 mg/L and total PCB content between 550 and 826 mg/L. These results correspond to the maximum of the response surface of the experimental design shown in Figure 6.

To determine the cell density of the *Nostoc* sp. species in the culture medium exposed to the photocatalytic effluent, the relationship between the absorbance at 665 nm and the cell count in the Neubauer chamber was used. In this way, it was established that, after the exposure time of 7 days, the *Nostoc* sp. species had the cell densities reported in Table 2.

The results presented in Table 2 show that the content of chlorophyll in the control medium for a photoperiod of 12 h/12 h more than twofold exceeded the content of chlorophyll obtained in the control medium for a photoperiod of 24 h/24 h, suggesting that the ideal photoperiod for the growth of the species is 12 h of light followed by 12 h of darkness. Additionally, for the control experiments, the cell density and the content of chlorophyll were notably higher than for the cultures exposed to the photocatalytic effluent; in this way, it was possible to conclude that the concentration of PCBs progressively inhibited the growth of the *Nostoc* sp. species, with the least cell growth being observed in the medium with the highest concentration of PCBs.

## 4. Conclusions

Photocatalytic treatment of a dielectric oil sample in an aqueous emulsion was able to achieve PCB removals close to 90%; additionally, the optimal conditions for the proposed experimental design were determined.

After analyzing the behavior of the total organic carbon content removal as a function of time, TOC removals close to 80% were established in a reaction time of 4 h. A van’t Hoff-type kinetic adjustment was performed, establishing pseudo-third-order reaction kinetics; however, the results suggest that the kinetics of this reaction is more complex and may involve different stages and reaction steps.

When carrying out the variation of the photoperiod for the growth of the *Nostoc* sp. species in a modified BG11 culture medium without an organic carbon source or trace metal solution, it was possible to establish that there were higher cell growth indicators when using 12 h of light followed by 12 h of darkness. Additionally, when subjecting the culture of the *Nostoc* sp. species to the exposure of the photocatalytic effluent to optimal conditions, it was determined that the concentration of PCBs progressively inhibited the growth of the species; the lowest cell growth was observed in the medium with the highest concentration of PCBs, possibly due to the fact that the presence of this type of substance generated a toxic effect on *Nostoc* sp. during acute exposure, with significant alterations in cell density and growth rates.

## Figures and Tables

**Table 1:** PCB species identified in the dielectric oil employed in this work.

Peak No.	Retention Time (min)	Area (%)	Compound	ID Factor	Formula
1	16.67	0.33	1,1′-Biphenyl, 2-chloro-	86	C_12_H_9_Cl
2	19.67	3.81	1,1′-Biphenyl, 2,2′-dichloro-	86	C_12_H_8_Cl_2_
3	21.28	0.48	1,1′-Biphenyl, 2,6-dichloro-	80	C_12_H_8_Cl_2_
4	21.95	1.16	2,6-Dichloro-1,1′-Bifenilo 1,1′-Biphenyl, 2,6′-dichloro-	77	C_12_H_8_Cl_2_
5	22.45	11.29	2,3-Dichlorobiphenyl	91	C_12_H_8_Cl_2_
7	25.64	16.45	2,4,6-Trichlorobiphenyl	91	C_12_H_7_Cl_3_
8	26.13	2.27	2,6-Dichlorobiphenyl	79	C_12_H_8_Cl_2_
10	27.12	5.37	2,2′,5-Trichloro-1,1′-biphenyl	84	C_12_H_7_Cl_3_
11	29.16	1.06	2′,3,4-Trichloro-1,1′-biphenyl	80	C_12_H_7_Cl_3_
12	30.12	24.02	3,4,4′-Trichloro-1,1′-biphenyl	94	C_12_H_7_Cl_3_
13	30.88	7.58	2,4,6-Trichloro-1,1′-biphenyl	88	C_12_H_7_Cl_3_
14	31.67	2.48	2,4,6-Trichloro-1,1′-biphenyl	83	C_12_H_7_Cl_3_
15	33.01	9.73	Methyl nonanoate	90	C_10_H_20_O_2_
16	33.74	2.88	2,2′,5,6-Tetrachloro-1,1′-biphenyl	82	C_12_H_6_Cl_4_
17	34.12	1.53	2,2′,4,5′-Tetrachloro-1,1′-biphenyl	77	C_12_H_6_Cl_4_
18	34.33	0.94	2,2′,6,6′-Tetrachloro-1,1′-biphenyl	73	C_12_H_6_Cl_4_
19	35.82	2.41	2,2′,4,5′-Tetrachloro-1,1′-biphenyl	79	C_12_H_6_Cl_4_
21	36.52	0.62	2,4,6-trichloro-1,1′-biphenyl	80	C_12_H_7_Cl_3_
23	37.28	0.73	2,2′,6,6′-Tetrachloro-1,1′-biphenyl	82	C_12_H_6_Cl_4_

**Table 2:** Results for the algae treatment of PCBs photocatalytic effluent.

Experiment	Initial PCBs (mg/L)	Photoperiod (Light Hours/Dark Hours)	Cellular Density (Cells/mL)	Chlorophyll Content (µg/L)	Growth Inhibition %
1	0	12/12	1.25 × 10^6^	5.88	N.A.
2	2.0	12/12	3.09 × 10^5^	1.49	74.7
3	5.0	12/12	N.A.	0.02	99.6
4	0	24/24	5.12 × 10^5^	2.27	87.6
5	2.0	24/24	4.02 × 10^4^	0.28	92.7
6	5.0	24/24	1.32 × 10^4^	0.16	74.7

## Data Availability

The reported results are archived in the institutional repository of Universidad Nacional de Colombia and can be seen at https://repositorio.unal.edu.co/handle/unal/1/browse?type=author&value=Camargo+Moreno%2C+Carlos+Ernesto. (accessed on 20 September 2020).
